# Combined lateral meniscus posterior root and meniscofemoral ligament injuries increase tibiofemoral forces and compromise rotational stability in ACL‐deficient and reconstructed knees: A systematic review and meta‐analysis of biomechanical studies

**DOI:** 10.1002/jeo2.70227

**Published:** 2025-04-03

**Authors:** Lika Dzidzishvili, Andrew S. Bi, Marko Ostojic, Jorge Chahla

**Affiliations:** ^1^ European Society of Sports Traumatology, Knee Surgery, Arthroscopy (ESSKA), Basic Science Committee Luxembourg Luxembourg; ^2^ Department of Orthopaedic Surgery and Traumatology, Knee Surgery and Arthroscopy Unit, Hospital Universitari Germans Trias i Pujol Universidad Autónoma de Barcelona Barcelona Spain; ^3^ Department of Orthopaedic Surgery Rush University Medical Center, Midwest Orthopedics at Rush Chicago Illinois USA; ^4^ Department of Sports Medicine and Knee, Orthopaedics and Traumatology Clinic University Hospital “Sisters of Mercy” Zagreb Croatia

**Keywords:** anterior cruciate ligament, anterior tibial translation, lateral meniscus posterior root, meniscofemoral ligament, rotational instability, tibiofemoral contact pressure

## Abstract

**Purpose:**

To compare biomechanical evidence on joint compression forces and rotational stability between isolated lateral meniscus posterior root (LMPR) tears and those with concurrent meniscofemoral ligament (MFL) injuries, with a secondary focus on assessing rotational stability in ACL‐deficient and ACL‐reconstructed knees.

**Methods:**

A comprehensive literature review was conducted following the 2020 PRISMA guidelines, using the Scopus, PubMed, and Embase databases from their inception through 7 October 2024. This review included biomechanical studies on healthy animal or human cadaveric knees that assessed lateral compartment contact area and peak pressure following isolated LMPR and MFL injuries, as well as kinematic outcomes in anterior cruciate ligament (ACL) deficient knees. The methodological quality of the studies was assessed using the Quality Appraisal for Cadaveric Studies (QUACS) scale.

**Results:**

Twelve studies involving 116 knees—86 human cadaveric and 30 porcine models—assessed tibiofemoral contact mechanics (contact area and pressure) and kinematic data. Both isolated LMPR tear and the combination of LMPR and MFL injuries significantly increased mean contact pressure in the lateral compartment compared to the intact state (*p* = 0.004 and <0.001, respectively). However, isolated LMPR tear did not significantly increase peak pressure in the lateral compartment (*p* = 0.55), whereas the combination with MFL injury caused a substantial rise (<0.001). LMPR repair restored both contact and peak pressures to levels that were not significantly different from those observed in the intact state (*p* = 0.86 and <0.28, respectively). Additionally, the combination of LMPR tear, MFL injury and ACL sectioning further increased anterior tibial translation (ATT) and internal tibial rotation (IR) during a simulated pivot shift test compared to isolated LMPR tear (<0.001). Although LMPR repair reduced rotational instability, it did not significantly restore ATT (*p* = 0.63) and IR (*p* = 0.923) during simulated pivot shifts in ACL‐reconstructed knees.

**Conclusions:**

A combined injury to the LMPR and MFL significantly increases mean and peak contact pressures in the lateral compartment compared to isolated LMPR tear and intact states, with LMPR repair restoring contact pressure to near‐normal levels. However, in knees with ACL deficiency or reconstruction, LMPR tear with MFL injury significantly increases IR during pivot shift testing, with LMPR repair unable to restore rotational stability to intact‐state levels.

**Level of Evidence:**

Level IV, systematic review of biomechanical studies.

**Abbreviations**
ACL: anterior cruciate ligament
ACLR: anterior cruciate ligament reconstruction
ANOVA: one‐way Analysis of Variance
ATT: anterior tibial translation
ER: external rotation
HSD: Tukey's Honestly Significant Difference
IQR: interquartile range
IR: internal rotation
LMPR: lateral meniscus posterior root
MFL: meniscofemoral ligament
MMPR: medial meniscus posterior root
N: Newton
N‐m: Newton metre
Pa: Pascal
QUACS: Quality Appraisal for Cadaveric Studies

## INTRODUCTION

Lateral meniscus posterior root (LMPR) tears occur in up to 14% of patients with concurrent anterior cruciate ligament (ACL) injuries [9, 16]. Biomechanical studies have demonstrated that the LMPR attachment plays a crucial role in stabilising the ACL‐deficient knee, especially by limiting excessive anterior tibial translation (ATT) and internal tibial rotation (IR) [10, 27].

Distinct biomechanical and clinical differences between lateral and medial posterior root attachments have been linked to varying clinical outcomes, which are widely explored in the literature [5, 18]. One of the unique biomechanical characteristics of the lateral roots is the presence of meniscofemoral ligaments (MFLs), which appear to stabilise the LMPR by counteracting the adverse effects of mechanical forces on the lateral compartment following an LMPR tear [4, 7]. This stabilising role of the MFL has become an emerging focus in meniscal root preservation surgery.

Research suggests that meniscal function may be preserved in LMPR tears if the MFL remains intact, as evidenced by tibiofemoral joint contact area and pressures in the lateral compartment [7]. Moreover, the presence of the MFL is increasingly recognised as a protective factor against meniscal extrusion (ME) [4, 6, 24]. Absence of the MFL appears more common in cases of extruded menisci with LMPR tear, suggesting that the MFL may play a role in mitigating ME, which in turn has implications for cartilage health and osteoarthritis (OA) progression [4, 24]. This may partly explain why LMPR tears typically exhibit lower rates of ME and OA progression compared to their medial counterparts [5].

Given the limited clinical or biomechanical evidence that specifically analyse LMPR tears with and without MFL injury, the primary purpose of this study is to synthesise biomechanical evidence on joint compression forces between isolated LMPR tear and those with concomitant MFL injuries. The secondary objective of the study included analysis of a subset of studies that examine ACL sectioning and reconstruction to evaluate the kinematics of ATT and IR in knees with isolated LMPR tears and combined MFL injuries. The authors hypothesized that combined injuries to the LMPR and MFL will significantly increase joint compression forces and reduce rotational stability in ACL deficient or reconstructed knees compared to isolated LMPR tear.

## METHODS

### Search criteria

The study adhered to the 2020 PRISMA (Preferred Reporting Items for Systematic Reviews and Meta‐Analyses) guidelines [23]. A comprehensive search was conducted on 7 October 2024, using the Scopus, PubMed, and Embase databases to identify literature on biomechanical outcomes related to isolated and combined LMPR and MFL injuries. The following search terms, combined with Boolean operators, were employed: ‘lateral meniscus posterior root tear’ AND ‘meniscofemoral ligament’; ‘meniscofemoral ligament’ AND ‘biomechanical study’; ‘meniscofemoral ligament’ AND ‘controlled laboratory study’; AND ‘meniscofemoral ligament’ AND ‘contact pressure’ OR ‘peak pressure’ OR ‘anterior tibial translation’ OR ‘internal tibial rotation’.

### Inclusion and exclusion criteria

Study inclusion criteria were as follows: (1) biomechanical studies conducted on healthy animal or human cadaveric knees in a controlled laboratory setting, assessing rotational instability or tibiofemoral contact mechanics (contact area and pressure) after combined LMPR tear and/or MFL injuries. (2) Studies reporting kinematic and joint compression forces in cases of isolated and combined LMPR and MFL ligament tears. Exclusion criteria included: (1) Biomechanical studies that did not evaluate or report kinematic outcomes following isolated or combined MFL injury. (2) Studies involving specimens with pre‐existing chondral or meniscal injuries or bone abnormalities. (3) Editorials, surveys, letters to the editor, and expert reviews.

### Data extraction and outcome measures

A customised data extraction spreadsheet was created to record all relevant information from the included studies. The extracted data were analysed qualitatively and quantitatively based on their methods, results, discussions, and conclusions. For studies that did not report data in a numeric format, values were approximated from graphs using graph digitisation tools. Additionally, for values reported as median and range, the mean and standard deviation were estimated using the methods outlined by Hozo et al. [15] The primary biomechanical outcome measures evaluated in this systematic review were mean contact and peak pressure in the lateral compartment at full extension and at 90° of knee flexion after isolated LMPR tear and combined LMPR and MFL injuries. For studies that included ACL sectioned and reconstructed knees, kinematic analysis of ATT and IR were compared during a simulated pivot shift manoeuvre with valgus torque applied 15° and 30° of knee flexion, as well as ATT under an anterior drawer force at 30° and 60° of knee flexion.

### Risk of bias assessment

Two investigators (L.D. and M.O.) independently screened articles by title, abstract, and full text. Risk of bias was assessed using the 13‐item Quality Appraisal for Cadaveric Studies (QUACS) scale, with items scored as 1 (yes/present) or 0 (no/absent) [30]. An ideal score is 13, with scores below 10 indicating a high risk of bias [30]. Any disagreement between the investigators was resolved by review of a third investigator (A.S.B).

### Statistical analysis

Continuous variables for each group were summarised using means, standard deviations, and ranges, while pairwise comparisons between groups were conducted with independent two‐sample t‐tests. Categorical variables were summarised as frequencies and proportions, with group comparisons assessed using chi‐square tests. For multi‐group comparisons of mean tibiofemoral contact pressure, ATT, and IR under valgus torque, a one‐way analysis of variance (ANOVA) was employed. When ANOVA indicated significant differences, pairwise comparisons were further examined using post‐hoc Tukey's Honestly Significant Difference (HSD) test to identify specific differences between groups. To visually represent group comparisons, box plots were generated for each variable, with each box depicting the interquartile range (IQR) and a horizontal line indicating the median. Whiskers extend up to 1.5 times the IQR or the furthest data points within that range, while outliers are displayed as individual points beyond the whiskers. To mitigate the risk of Type I error associated with multiple comparisons, *p*‐values were adjusted using the Bonferroni correction. All statistical analyses were conducted using R (version 4.3.3, R Core Team, 2024).

## RESULTS

### Study overview and reported biomechanical data

**Figure 1:**
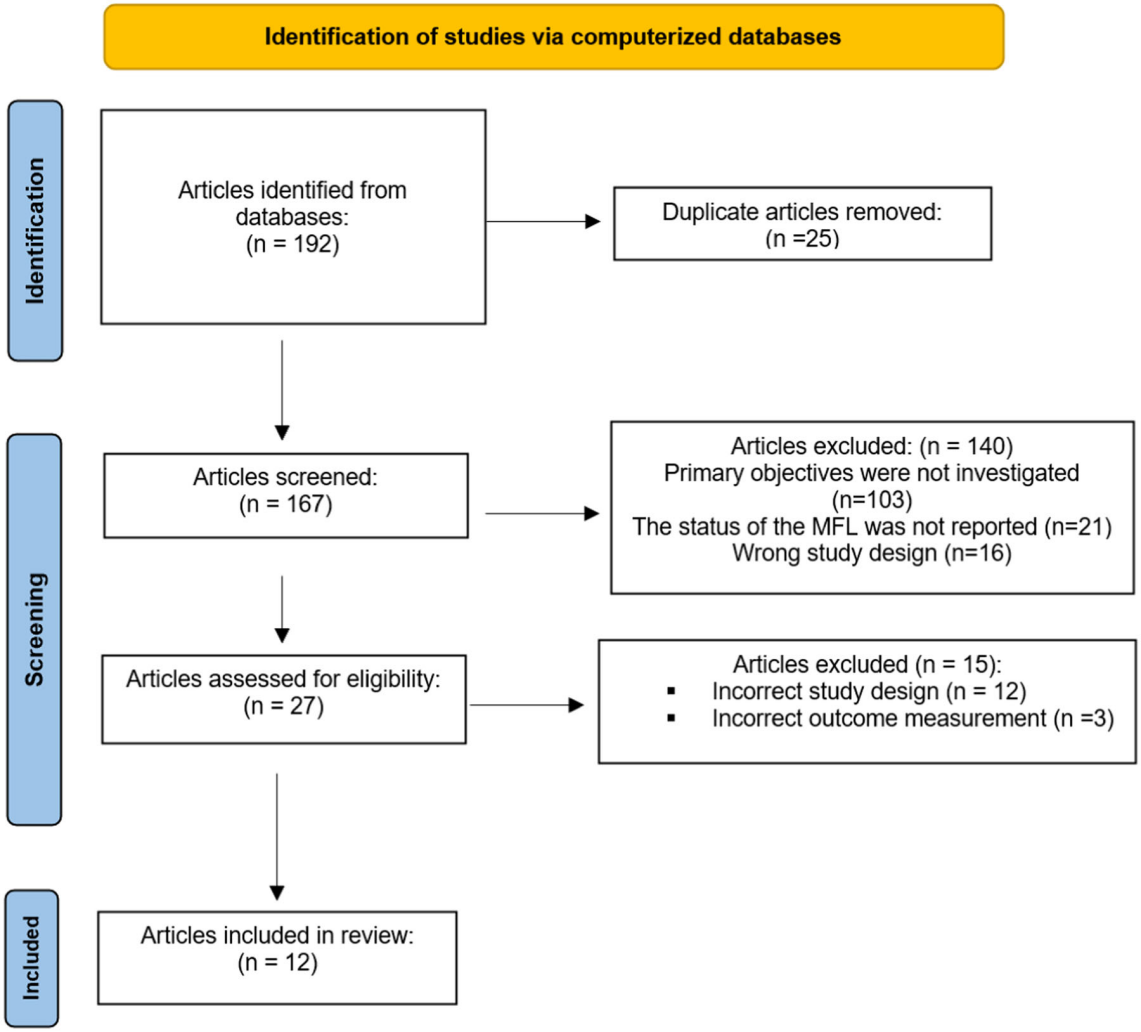
PRISMA (Preferred Reporting Items for Systematic Reviews and Meta‐Analyses) flowchart.

Twenty‐seven full‐text articles were reviewed, with fifteen studies excluded based on eligibility criteria. Twelve biomechanical studies met the inclusion criteria and were included in this investigation (Figure 1). All studies were controlled laboratory experiments conducted on fresh‐frozen human cadaveric or porcine knee specimens, encompassing a total of 116 specimens—86 human cadaveric knees and 30 porcine knees. Four studies solely investigated isolated LMPR tear with or without MFL injuries [2, 7, 21, 26], while eight studies investigated LMPR tears +/‐ MFL injuries in the setting of ACL section or reconstruction [8, 10, 11, 12, 25, 27, 28, 29]. Detailed characteristics are presented in Table 1. Due to the novelty and emerging significance of the MFL in biomechanical research, there are only a limited number of studies that analyse kinematic data at various knee flexion angles during testing. Consequently, some studies were referenced more extensively due to their comprehensive datasets. Most studies provided data across a range of knee flexion angles (0°–90°). Data were insufficient for pooling at each specific tested angle. However, efforts were made to aggregate and present clinically relevant findings wherever possible.

**Table 1:** Study characteristics and testing conditions of the included biomechanical studies.

First author (year)	*N* and origin of specimens (mean age; range)	Testing conditions	Applied loading force and the tested knee flexion angles	Main findings
Forkel et al. [7] (2013)	10 Porcine	(1) Intact, (2) LMPR tear + MFL tear, (3) Isolated LMPR tear; (4) isolated MMPR repair	Axial load (100 N) at 30° of flexion.	After additional cutting of the MFL, the tibiofemoral contact pressure was significantly higher in comparison with the intact state.
Frank et al. [11] (2017)	10 Human (57; 49–62)	(1) LMPR tear, (2) ACL cut (3) LMPR tear + MFL tear + ACL cut	Simulated pivot‐shift test (5‐Nm IR torque combined with a 10‐Nm valgus torque) at 0°, 20°, 30°, 60° and 90° of knee flexion; an anterior translation load (88 N) at 0°, 30°, 60° and 90° of knee flexion; and IR (5 Nm) at 0°, 30°, 60°, 75° and 90°.	Cutting the LMPRT and MFLs significantly increased ATT during a pivot‐shift test at 20° and 30° when compared with the ACL‐cut state.
Amadi et al. [2] (2008)	5 Human (66–80)	Isolated MFL tear	Axial load (50 N to 1800 N) at full extension +/‐5 N‐m IR torque.	The MFLs play a role in reducing lateral tibiofemoral contact pressure.
Shybut et al. [27] (2015)	8 Human (44; 25–63)	LMPR tear+MFL + ACL tear	Anterior load (90 N) at 15°, 30°, 45°, 60° and 90°), simulated pivot‐shift IR (3 Nm), and valgus moment (5 and 7 Nm) during knee flexion from 15° to 60°.	LMPRT and injury to the MFL resulted in significantly increased instability in ACL deficient knees during a simulated pivot shift.
Ohori et al. [21] (2019)	20 Porcine	MFL tear, isolated LMPR tear, and LMPR + MFL tears	Axial load (250 N) and valgus torque (5 N‐m) at 30°, 60° and 90° of knee flexion.	The MFL and LMPR functioned reciprocally. The in‐situ force of the LM decreased only after resecting both the MFL and LMPR under a compressive load and valgus torque.
Sequeira et al. [26] (2023)	10 Human (77.2; 58‐94)	(1) Intact; (2) LMPR tear (25%); (3) LMPR tear (50%); (4) LMPR tear (75%); (5) LMPR tear (100%); (6) LMPR tear (100%) + MFL tear;	Axial load (100 to 1000 N) at 0°, 30°, 45°, 60° and 90° of knee flexion.	Progressive radial tears of the LMPRTs were not associated with an increase in tibiofemoral contact pressure or decrease in lateral compartment surface area. LMPRT + MFLT were associated with increased joint contact pressure and decreased lateral compartment surface.
Geeslin et al. [12] (2015)	10 Human (57; 43–65)	(1) Intact; (2) LMPR tear; (3) LMPR + MFL tears; (4) LMPR + MFL + ACL tears;(5) LMPR tear + MFL tear + ACLR;(6) LMPR repair + MFL tear + ACLR	Axial loading (1000‐N) at 0°, 30°, 45°, 60° and 90° knee flexion.	LMPRT and MFLT resulted in significantly decreased lateral compartment contact area and increased pressure compared to the intact state, whereas a LMPRT with intact MFLs did not significantly affect contact mechanics.
Forkel et al. [8] (2014)	10 Human (68.7; 61–79)	(1) Intact, (2) Isolated LMPR tear; (3) LMPR tear + MFL tear; (4) LMPR repair using anatomic transosseous tunnel; (5) LMPR repair using a tibial ACL tunnel.	Axial loading (100 N) at 0° and 90° of knee flexion.	After transection of the LMPR the contact pressure did not increase significantly. The additional transection of the MFL led to a significant increase in the contact pressure.
Forkel et al. [10] (2018)	8 Human (62; 54–70)	(1) Intact, (2) ACL cut, (3) ACL cut + LMR tear, (4) ACL cut + LMPR tear + MFL tear; (5) ACL cut + LMPR repair	Axial load (20 N), internal torque (5 N), anterior translation (50 N)	LMPRT and an additional tear of the MFL contribute to an increase of instability of the ACL‐deficient knee.
Seiter et al. [25] (2023)	10 Human	(1) Intact; (2) ACLR (3) LMPR tear; (4) LMPR repair, and (5) MFL augmentation.	Axial load (20‐N). (1) 88‐N Anterior drawer, (2) 5‐N‐m IR, (3) 5‐Nm ER, (4) 5‐Nm Varus, and (5) 5‐N‐m Valgus at 0° and 30° of knee flexion. A simulated pivot shift test (5‐N‐m IR, 5‐N‐m varus, and 88‐N anterior load) at 30° of flexion.	LMPRT may place the ACLR knee at risk due to significantly increased ATT in the simulated pivot shift. LMPRR with and without MFL augmentation restored ATT.
Tang et al. [28] (2019)	13 Human (47; 32–59)	(1) Intact; (2) ACLR (3) ACLR + LMPR tear+ MFL tear; (4) ACLR + LMPR repair.	An 89‐N anterior tibial load applied at 0°, 15°, 30°, 60° and 90° of knee flexion; (2) a combined 7‐N‐m valgus and 5‐N‐m internal tibial torque (simulated pivot‐shift test) applied at 0°, 15° and 30° of knee flexion.	LMPRT after ACLR led to an increase in ATT and the LMPRR reduced anterior laxity under anterior tibial loading at 15° and 30° of flexion.
Uffmann et al. [29] (2021)	12 Human (18‐60)	(1) Intact; (2) ACLR (3) ACLR + LMPR tear + MFL tear; (4) ACLR + LMPR repair	An 88‐N anterior drawer force, internal and external torque of 5‐N‐m applied at 0°, 15°, 30°, 60° and 90° of flexion. A simulated pivot shift was applied at 0°, 15° and 30° of flexion.	LMPRT + MFLT increases rotational and anterior laxity of the knee and places increased strain across reconstructed ACL grafts. Subsequent root repair did not result in a statistically significant reduction in strain.

Abbreviations: ACL, anterior cruciate ligament; ACLR, anterior cruciate ligament reconstruction; ER, external rotation; IR, internal tibial rotation; LMPR, lateral meniscus posterior root; MFL, meniscofemoral ligament; MMPR, medial meniscus posterior root; N, Newton; N‐m, Newton metre; Pa, Pascal.

When numerical data were unavailable, computer vision‐assisted software was utilised to extract numerical values from graphs and plots. Tibiofemoral contact mechanics assessed in this review included both average and peak pressures in the lateral compartment. Given the frequent association of LMPR tear with ACL‐deficient knees, kinematic data were also analysed. As the pivot shift test is clinically significant and typically performed at lower flexion angles, anterior ATT and tibial IR under valgus torque were analysed at 15° and 30°. Additionally, ATT was assessed under anterior loading. The impact of isolated LMPR tear and combined LMPR tear with MFL injury in ACL reconstructed knees during anterior drawer testing was also evaluated.

### Literature quality assessment

**Figure A1:**
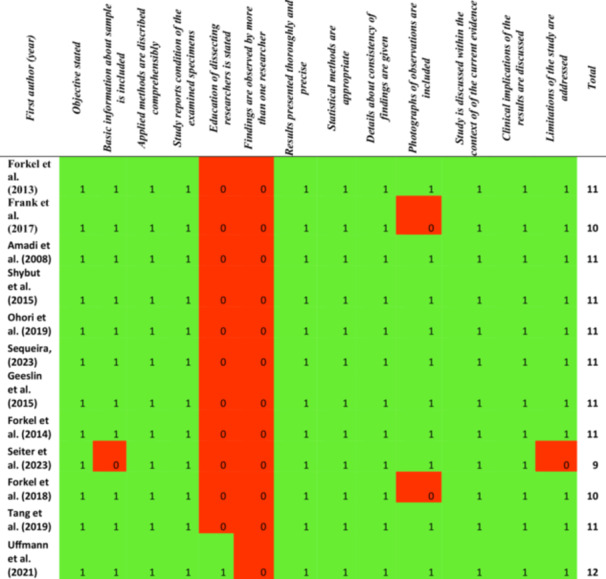
Quality Appraisal for Cadaveric Studies (QUACS) bias analysis.

Bias analysis of the 12 biomechanical studies was performed using the QUACS bias analysis tool (Appendix Figure A1). The mean score was 10.8 ± 0.8 (range, 9–12).

### Peak contact pressure in the lateral compartment

**Figure 2:**
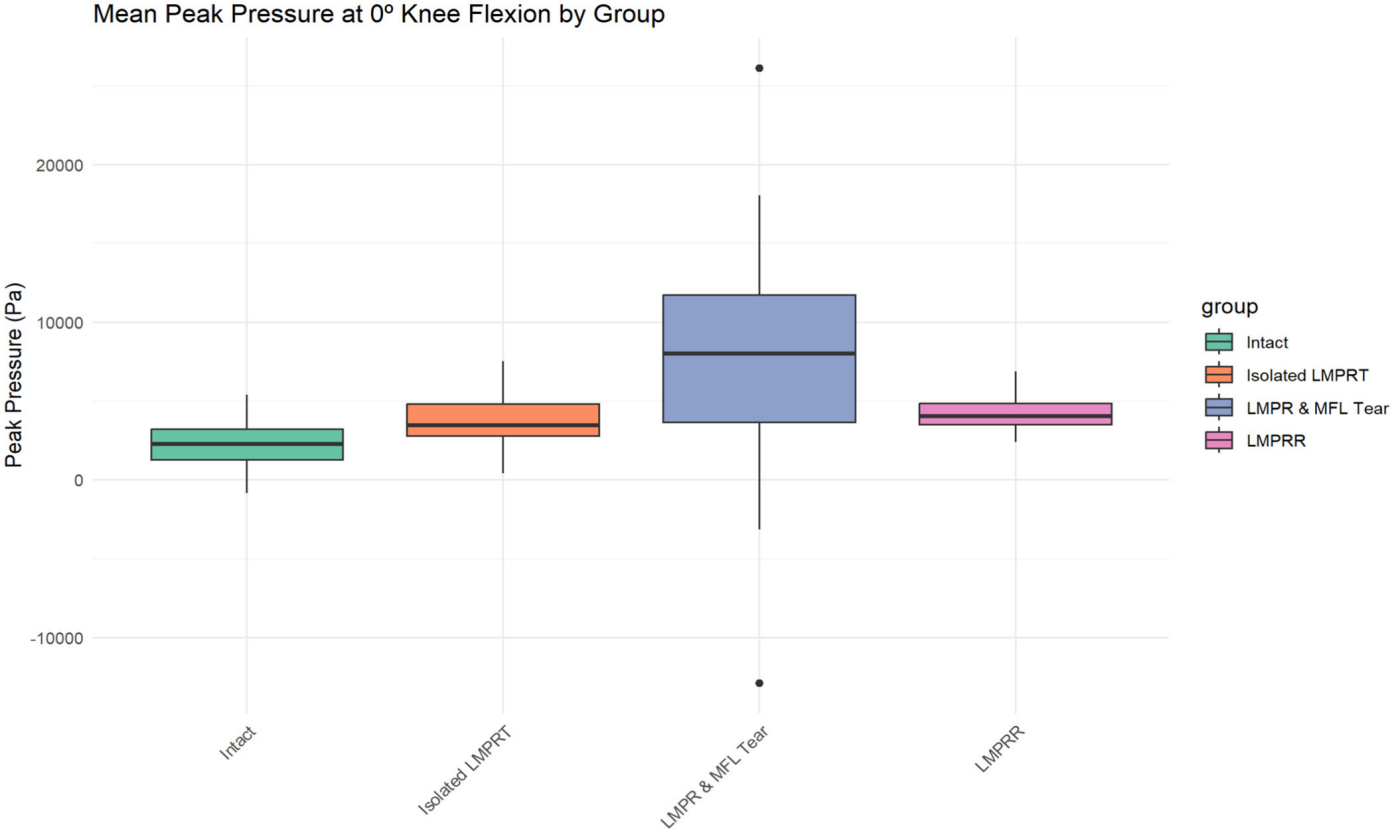
The box plot shows the distribution of mean peak pressure in lateral compartment at full extension across four experimental conditions: Intact, isolated LMPR tear, combined LMPR and MFL injuries, and after LMPR repair. Each box represents the interquartile range (IQR), with a horizontal line indicating the median. Whiskers extend up to 1.5 times the IQR or the furthest data points within that range, while outliers are displayed as individual points beyond the whiskers. ACL, anterior cruciate ligament; LMPR, lateral meniscus posterior root; LMPRT, lateral meniscus posterior root tear; LMPRR, lateral meniscus posterior root repair; MFL, meniscofemoral ligament.

Four studies [2, 7, 8, 12] investigated peak contact pressures in the lateral tibiofemoral compartment. Isolated LMPR tear resulted in a non‐significant increase in peak pressure compared to the intact state. However, when LMPR tear was accompanied by concurrent damage to the MFL, there was a significant increase in peak pressure in the lateral compartment compared to both the isolated LMPR tear and intact states. Furthermore, the repair of LMPR tear restored peak pressure to levels that were not significantly different from those observed in the intact state (Table 2 and Figure 2).

**Table 2:** Pairwise comparisons of peak pressure in the lateral compartment at 0° of knee flexion using Tukey's HSD post hoc test.

Testing conditions	Mean difference*	Lower bound#	Upper bound^	*p*‐Adj"
Isolated LMPR tear vs. Intact	1382.6	−1371.2	4136.4	0.556
LMPR and MFL tear vs. Intact	5662.8	2908.9	8416.7	**<0.001**
LMPR repair vs. Intact	1886.3	−867.5	4640.2	0.285
LMPR and MFL tear vs. Isolated LMPR tear	4280.2	1526.3	7034.1	**0.0005**

*Note*: Significant *p* values are shown in bold.

Abbreviations: ACL, anterior cruciate ligament; LMPR, lateral meniscus posterior root; MFL, meniscofemoral ligament.

* The estimated difference in mean values between each pair of groups. A positive value indicates that the mean of the first group in the pair is higher than the second group, while a negative value indicates the opposite.

^#^ Represents the lower bound of the 95% confidence interval for the mean difference. This indicates the minimum likely difference between the groups at a 95% confidence level.

^^^ Represents the upper bound of the 95% confidence interval for the mean difference. This represents the maximum likely difference between the groups at a 95% confidence level.

^"^ The adjusted *p* value for each pairwise comparison, corrected for multiple testing.

### Mean contact pressures in the lateral compartment

**Figure 3:**
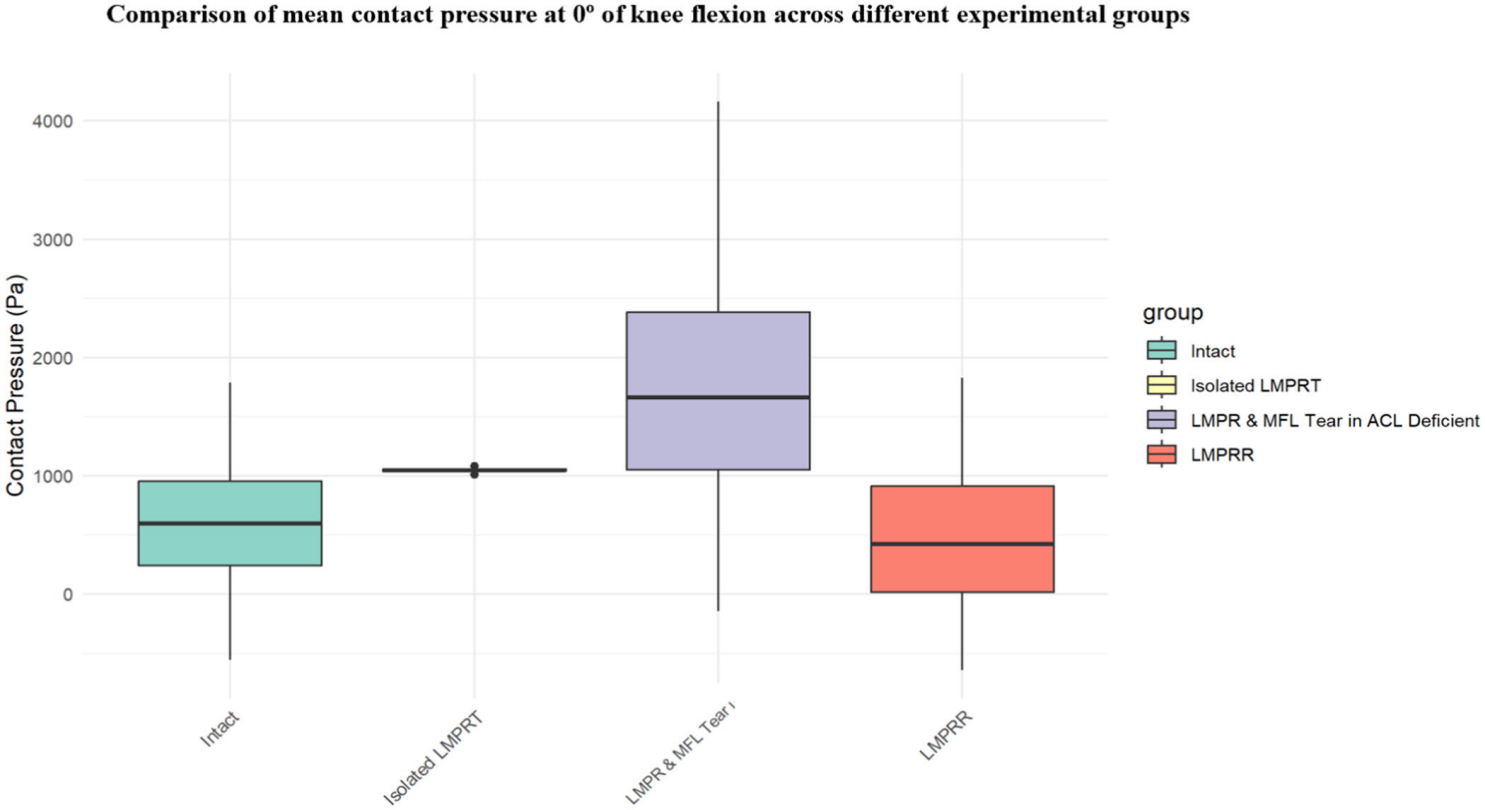
The box plot displays the distribution of peak pressure in the lateral compartment at full extension across four experimental conditions: Intact, isolated LMPR tear, combined LMPR and MFL injuries, and after LMPR repair. Each box represents the interquartile range (IQR), with a horizontal line marking the median value. Whiskers extend to 1.5 times the IQR or the most extreme data points within this range, while outliers are shown as individual points beyond the whiskers. ACL, anterior cruciate ligament; LMPR, lateral meniscus posterior root; LMPRT, lateral meniscus posterior root tear; LMPRR, lateral meniscus posterior root repair; MFL, meniscofemoral ligament.

Five studies reported the mean contact pressure in the lateral compartment [2, 7, 8, 12, 26]. Isolated LMPR tear led to a significant increase in mean contact pressure in the lateral tibiofemoral compartment at both 0° and 90° of knee flexion compared to the intact state. Mean contact pressure was again significantly higher with combined LMPR and MFL injuries compared to the isolated LMPR tear state. Conversely, LMPR repair significantly reduced the contact pressure in the lateral compartment, showing no statistically significant difference from the intact condition (Table 3 and Figure 3).

**Table 3:** Pairwise comparisons of mean contact pressure in the lateral compartment at 0° of knee flexion using Tukey's HSD post hoc test.

Testing conditions	Mean difference*	Lower bound#	Upper bound^	*p*‐adj"
Isolated LMPR tear vs. Intact	453.6	105.8	801.4	**0.004**
LMPR and MFL tear vs. Intact state	1194.9	817.6	1572.2	**<0.001**
LMPR repair vs. Intact	−100.8	−438.3	236.5	0.865
LMPR and MFL tear vs. Isolated LMPR tear	741.2	354.6	1127.8	**<0.001**
LMPR repair vs. Isolated LMPR tear	−554.5	−902.3	−206.6	**0.0003**
LMPR repair vs. LMPR and MFL tear	−1295.8	−1673.1	−918.5	**<0.001**

*Note*: Significant *p* values are shown in bold.

Abbreviations: ACL, anterior cruciate ligament; LMPR, lateral meniscus posterior root; MFL, meniscofemoral ligament.

* The estimated difference in mean values between each pair of groups. A positive value indicates that the mean of the first group in the pair is higher than the second group, while a negative value indicates the opposite.

^#^ Represents the lower bound of the 95% confidence interval for the mean difference. This indicates the minimum likely difference between the groups at a 95% confidence level.

^^^ Represents the upper bound of the 95% confidence interval for the mean difference. This represents the maximum likely difference between the groups at a 95% confidence level.

^"^ The adjusted *p* value for each pairwise comparison, corrected for multiple testing.

### Pivot‐shift testing

**Figure 4:**
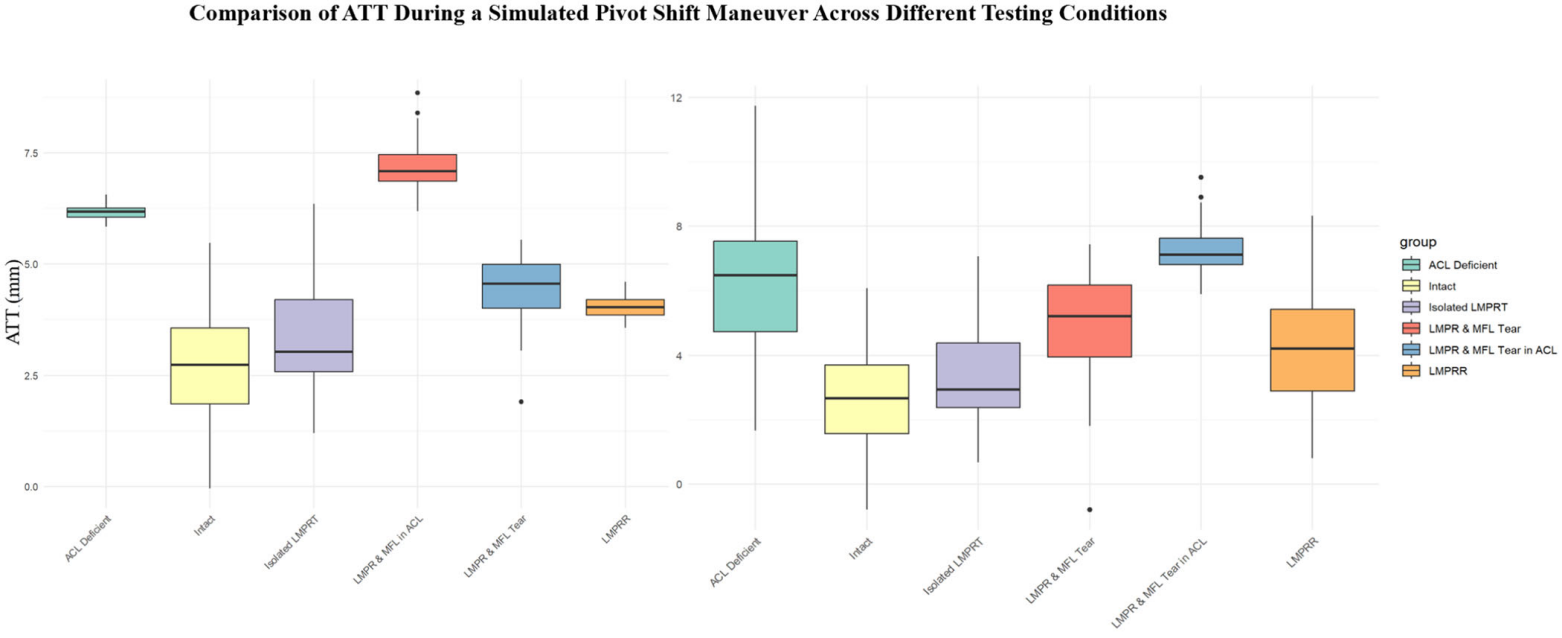
The box plot shows the distribution of mean ATT during simulated pivot shift test at 20° (left) and 30° (right) knee flexion across experimental conditions: Intact, ACL deficient, isolated LMPR tear in an ACL‐deficient knee, combined LMPR and MFL injuries in an ACL‐deficient knee, and LMPR repair. ACLR: ACL reconstruction state, ACLR + LMPR + MFL tear: ACL reconstruction with combined LMPR, and MFL tear, ACLR + LMPR tear: ACL reconstruction with LMPRR. ACL, anterior cruciate ligament; ATT, anterior tibial translation; LMPR, lateral meniscus posterior root; MFL, meniscofemoral ligament.

Seven studies reported on ATT during simulated pivot shift testing with a combination of LMPR ± MFL injuries, with ACL sectioning and subsequent reconstruction [8, 11, 21, 25, 27, 28, 29]. The mean ATT in the intact state was 2.8 ± 1.5 mm, which increased non‐significantly to 3.1 ± 1.5 mm in knees with isolated LMPR tear (Table 4 and Figure 4 **).** In knees with additional MFL injury, the mean ATT was 4.0 ± 2.4 mm, which was significantly higher than that observed in both the intact state and the isolated LMPR tear group (Table 4). Knees with both LMPR and MFL injuries in an ACL‐deficient state exhibited an ATT of 7.3 ± 0.7 mm during the simulated pivot shift test at 20° of flexion. This value was significantly higher than those in the intact state, the isolated LMPR tear, isolated ACL injury, and the combined LMPR and MFL injuries in ACL‐intact states (Table 4 and Figure 4).

**Table 4:** Pairwise comparisons between groups using Tukey HSD post hoc test for ATT during simulated pivot shift test.

Testing conditions	Mean difference*	Lower bound#	Upper bound^	*p*‐adj"
Intact vs. ACL deficient	−3.4	−4.2	−2.6	**<0.001**
Isolated LMPR tear vs. Intact	0.5	‐0.2	1.3	0.293
Isolated LMPR tear vs. ACL deficient	−2.9	−3.7	−2	**<0.001**
LMPR and MFL tears in ACL deficient knee vs. isolated ACL tear	1.1	0.2	2	**0.007**
LMPR and MFL tears vs. isolated ACL tear	−1.8	−2.6	−0.9	**<0.001**
LMPR and MFL tear vs. intact	1.643	0.897	2.4	**<0.001**
LMPR and MFL tear in ACL deficient knee vs. Intact	4.5	3.7	5.277	**<0.001**
LMPR and MFL tear vs. Isolated LMPR tear	1.1	0.3	1.9	**<0.001**
LMPR and MFL tears in ACL deficient knee vs. Isolated LMPR tear	3.9	3.1	4.8	**<0.001**
LMPR and MFL tears vs. LMPR and MFL tear in ACL deficient knee	−2.8	−3.7	−3	**<0.001**
LMPR repair vs. LMPR and MFL tears in ACL deficient knee	−3.2	−4	−2.4	**<0.001**
LMPR repair vs. LMPR and MFL tears	−0.4	−1.1	0.3	0.638
LMPR repair vs. Intact	1.3	0.6	1.9	**<0.001**
LMPR repair vs. Isolated LMPR tear	0.7	0.01	1.4	0.045

*Note*: Significant *p* values are shown in bold.

Abbreviations: ACL, anterior cruciate ligament; LMPR, lateral meniscus posterior root; MFL, meniscofemoral ligament.

* The estimated difference in mean values between each pair of groups. A positive value indicates that the mean of the first group in the pair is higher than the second group, while a negative value indicates the opposite.

^#^ Represents the lower bound of the 95% confidence interval for the mean difference. This indicates the minimum likely difference between the groups at a 95% confidence level.

^^^ Represents the upper bound of the 95% confidence interval for the mean difference. This represents the maximum likely difference between the groups at a 95% confidence level.

^"^ The adjusted *p* value for each pairwise comparison, corrected for multiple testing.

Notably, isolated LMPR repair reduced ATT but did not result in significant changes compared to the LMPR tear and MFL injuries or the intact state. Additionally, the combination of LMPR and MFL injuries in ACL‐deficient knees resulted in significantly increased ATT during the simulated pivot shift test compared to similar injury patterns in ACL‐intact and isolated LMPR tear states. Furthermore, the additional MFL injury in knees with LMPR and ACL‐deficiency further increased both ATT and IR during the simulated pivot shift test when compared to isolated LMPR tear with and without ACL injury (Table 4 and Figure 4).

**Figure 5:**
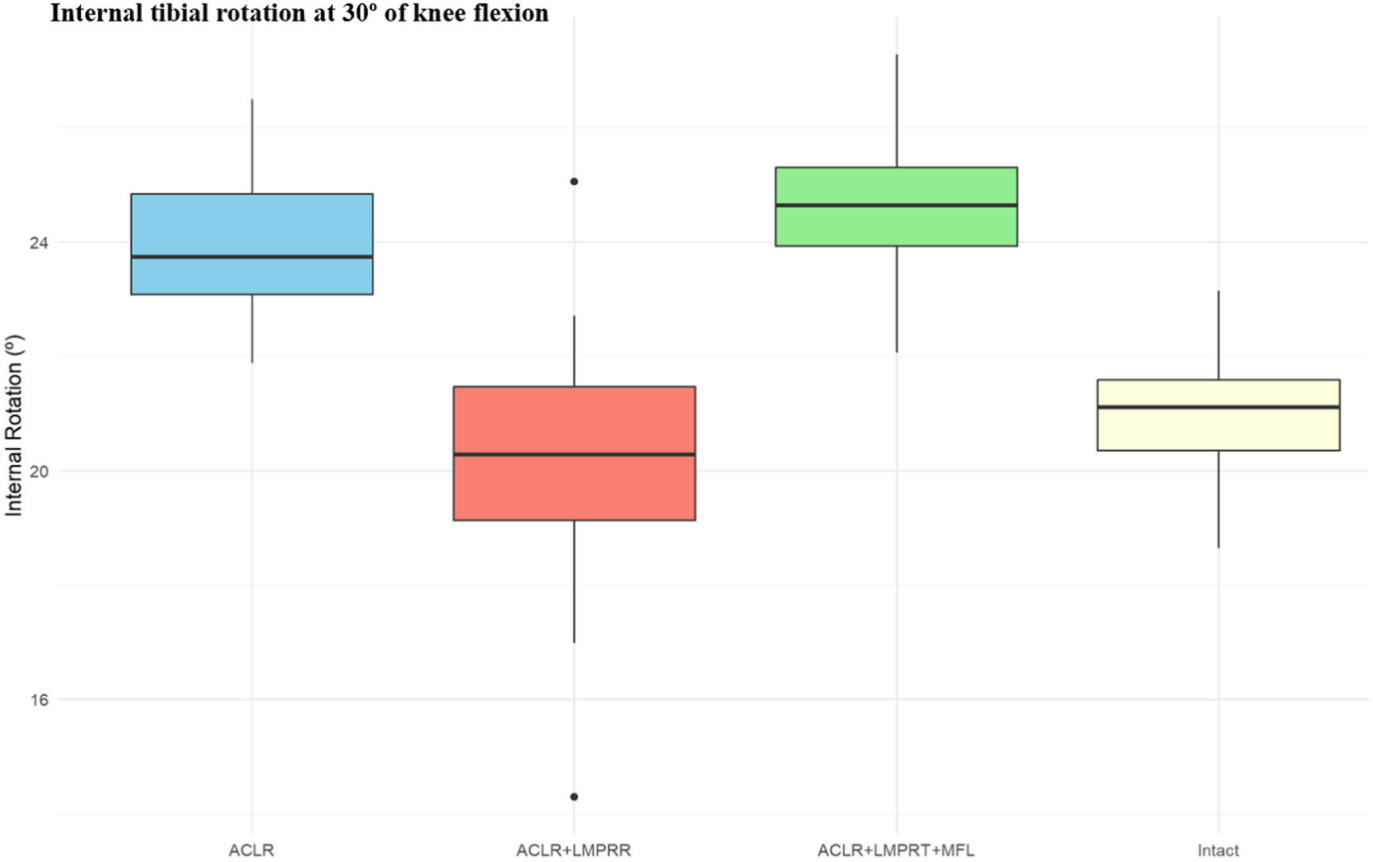
The box plot shows the distribution of mean IR (°) at 30° of knee flexion in ACL‐ reconstructed knees. ACLR, ACL reconstruction alone; ACLR + LMPR + MFL tear: ACL reconstruction with combined LMPR and MFL tear, and ACLR + LMPR tear: ACL reconstruction with LMPRR. ACL, anterior cruciate ligament; LMPR, lateral meniscus posterior root; MFL, meniscofemoral ligament.

Four studies reported on IR under valgus torque after combined LMPR and MFL injuries in the setting of ACL sectioning and reconstruction [10, 11, 25, 29]. In ACL‐deficient knees, combined sectioning of the LMPR and MFL significantly increased IR at both 30° and 90° of flexion angles compared to isolated LMPR tear (*p* < 0.001). In ACL‐reconstructed knees, a combined injury to the LMPR tear and MFL significantly increased IR at 30° of knee flexion (*p* = 0.007). Although LMPR repair reduced rotational instability, it did not significantly restore IR (*p* = 0.10) (Figure 5). The data available were insufficient to analyse IR at 90° of knee flexion in ACL‐reconstructed specimens.

### Anterior tibial translation under anterior drawer force application

**Figure 6:**
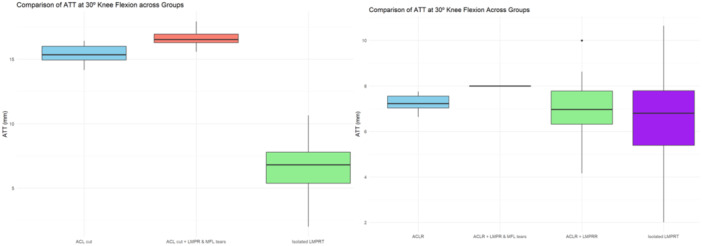
The box plot illustrates the distribution of mean ATT (mm) at 30° of knee flexion in ACL deficient (left) and reconstructed (right) knees under anterior drawer force across experimental states. Each box represents the interquartile range (IQR) with the horizontal line indicating the median value. Whiskers extend up to 1.5 times the IQR or to the furthest data points within that range, while individual points beyond the whiskers denote outliers. ACLR: ACL reconstruction, ACLR + LMPR and MFL tear: ACL reconstruction with combined LMPR and MFL tear, and ACLR + LMPR tear: ACL reconstruction with LMPRR. ACL, anterior cruciate ligament; ATT, anterior tibial translation; LMPR, lateral meniscus posterior root; MFL, meniscofemoral ligament.

Five studies reported on ATT under anterior drawer force [11, 21, 27, 28, 29]. The mean ATT at 30° in the intact state was 5 ± 1.6 mm, which increased to 6.5 ± 2.3 mm in knees with isolated LMPR tear. This increase was further elevated to 15.4 ± 0.7 mm in cases with root tears associated with ACL injury (*p* < 0.001). In ACL‐deficient knees with combined LMPR and MFL injuries, the mean ATT at 30° of knee flexion was 16.5 ± 0.6 mm, which was significantly greater than the ATT observed in isolated ACL tears (*p* = 0.02) (Figure 6). In ACL‐reconstructed knees with intact LMPR and MFL, the mean ATT at 30° of knee flexion was 7.3 ± 0.4 mm. This increased to 8 ± 0.1 mm with combined LMPR and MFL injuries (*p* = 0.28). Following root repair, the ATT decreased to 7.3 ± 0.3 mm, which was not significantly different from the ACL‐reconstructed state (*p* = 0.97) but remained significantly greater than the intact knee (*p* < 0.001; Figure 6).

### ACL graft strain during anterior drawer testing at 30° of knee flexion

Three studies reported the strain on the ACL graft during anterior drawer testing [25, 28, 29].

**Figure 7:**
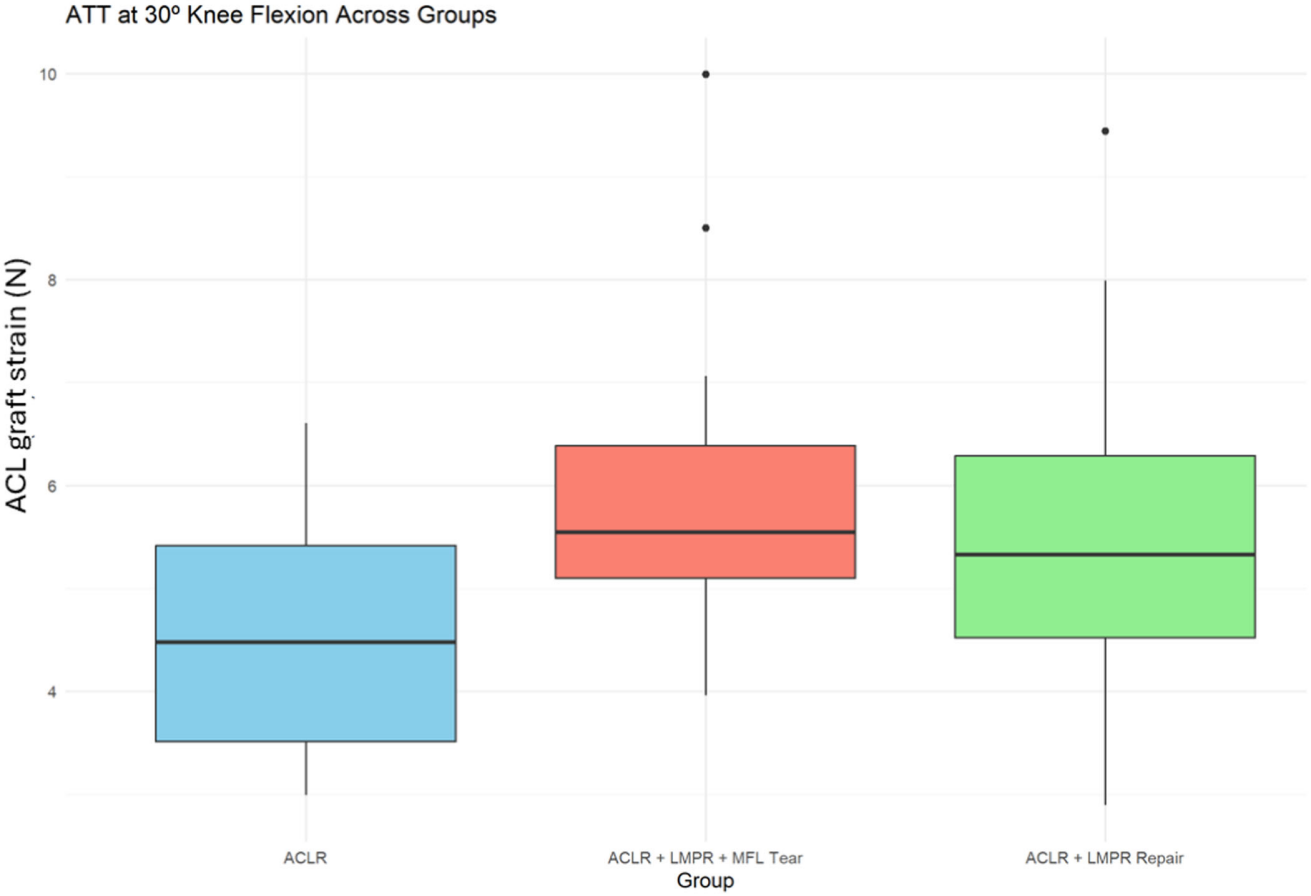
Box plot of ACL graft strain (N) across different experimental states during anterior drawer testing at 30° of knee flexion. ACLR: ACL reconstruction alone, ACLR + LMPR + MFL tear: ACL reconstruction with combined LMPR and MFL tear, and ACLR + LMPR tear: ACL reconstruction with LMPRR. ACL, anterior cruciate ligament; LMPR, lateral meniscus posterior root; MFL, meniscofemoral ligament.

Combined sectioning of the LMPR and MFL significantly increased ACL graft force during anterior drawer testing at 30° of knee flexion compared to the ACL reconstructed state with intact LMPR and MFL (*p* = 0.009). Furthermore, LMPR repair reduced graft strain to levels that were not significantly different from the ACLR state with an intact meniscus (*p* = 0.1) (Figure 7).

## DISCUSSION

The main finding of this systematic review of biomechanical studies is that combined injury to the LMPR and MFL significantly increases mean contact and peak pressures in the lateral compartment compared to an isolated LMPR tear, which maintains pressure levels closer to the intact state. Additionally, in knees with ACL deficiency or reconstruction, combined LMPR and MFL tears increase ATT with anterior drawer and IR with pivot shift, imposing greater load on the ACL graft than isolated ACL or LMPR tears.

The growing focus on meniscal root tears over the past decade has sparked increased interest in understanding the biomechanical role of the MFL. As clinical and radiological outcomes appear to differ between medial and lateral root tears, and as patient characteristics vary, the MFL has gained attention as a primary stabiliser of the LMPR attachment [5, 7]. Increased joint compression forces are known to correlate with root injuries and may lead to cartilage damage [1, 8, 12]. The current review found that combined LMPR and MFL injuries elevate both mean and peak contact pressures in the lateral compartment, whereas isolated LMPR tears with intact MFLs did not significantly impact contact mechanics. This finding aligns with previous research showing no significant difference in mean contact pressure between the intact state and an isolated LMPR tear [7]. Furthermore, the role of the MFL as a crucial stabiliser of the LMPR was highlighted in a previous biomechanical study that reported joint compression forces in the lateral and medial compartments following isolated lateral and medial root tears, respectively [7]. The study found that, after an induced isolated LMPR tear, mean pressure in the lateral compartment did not significantly increase compared to the intact state. In contrast, following a MMPR tear, joint compression forces in the medial compartment significantly increased relative to the intact knee [7].

Overall, two clinically relevant hypotheses emerge from the above findings. First, the available evidence suggests a potentially greater degree of load seen by the lateral compartment in combined LMPR and MFL injuries compared to isolated LMPR tears. Second, isolated MMPR tears may be more biomechanically deleterious than isolated LMPR injuries. This underscores the significant stabilising role of the MFL attachments in the lateral compartment and the higher incidence and severity of ME following MMPR tears compared to lateral root injuries. Future research should prioritise comparing joint compression forces in isolated MMPR tears versus combined LMPR and MFL injuries to further elucidate the biomechanical and clinical implications of these injuries.

It is well‐established that LMPR tears often occur concurrently with ACL tears [9]. In the present review, combined LMPR tear and MFL injuries significantly increased ATT and IR during a simulated pivot shift test in ACL‐deficient knees. This finding suggests that combined injuries in the context of an ACL tear may result in a higher grade of pivot shift. Additionally, the MFLs appear to contribute further to increased IR in ACL‐deficient knees under valgus torque. These findings are clinically relevant for several reasons. First, LMPR tears, which frequently accompany ACL injuries, are commonly observed in younger patients. The combined disruption of the LMPR and MFL may lead to increased contact stress and reduced rotatory stability, potentially increasing the load on an ACL reconstruction graft. In this study, significantly greater ACL graft force was observed during anterior drawer testing at 30° of knee flexion in cases of combined LMPR and MFL sectioning compared to the ACL‐reconstructed state with intact LMPR and MFL (*p* = 0.009).

Additionally, data from the current review showed that in ACL‐deficient knees, combined sectioning of the LMPR and MFL significantly increased IR at both 30° and 90° flexion angles compared to isolated LMPR tear or ACL cut states (*p* < 0.001). This aligns with previous biomechanical studies indicating the secondary rotational stability provided by the LMPR and that combined LMPR and MFL injuries in ACL‐deficient knees significantly increase IR [11]. Notably, although LMPR repair reduced rotational instability, it did not fully restore stability to the level of the intact state (*p* < 0.001). The lack of significant differences between combined LMPR tear and MFL injuries in ACL‐reconstructed knees and the isolated ACL reconstruction states may suggest that anteroposterior stability is primarily driven by the ACL, rather than the LMPR. In contrast, the LMPR and MFL act as significant secondary stabilisers to IR stability with the pivot shift mechanism, as evidenced by the significant increase in IR with LMPR tear and MFL injuries in ACL‐deficient states, which is restored with LMPR repair to a near‐intact state [11, 27, 28].

### Limitations

This study has several limitations. The primary limitation of the current review is the scarcity of evidence in the existing literature regarding the impact of the MFL in cases both with and without LMPR tears. Unfortunately, the available clinical data are insufficient to enable meaningful pooling or robust conclusions. Additionally, some studies fail to specify whether the MFL was injured in the included patients, a significant confounding factor identified by the authors. As such, these studies were excluded from the current review. Consequently, the analysis is limited to biomechanical investigations. Furthermore, the available studies conducted on human cadaveric knees are insufficient to derive robust and meaningful conclusions.

Another key limitation of the current review is the challenge of pooling data from studies that employ diverse methodologies and testing models. The inclusion of investigations using porcine knee models may raise concerns regarding the validity and generalisability of the results. However, the porcine knee is widely recognised in the literature as a valid model for biomechanical research [3, 17, 19, 20, 22]. While there are anatomical differences between porcine and human cadaveric knees, these differences are well‐documented [13]. For instance, quadruped knees have a thicker MFL and a relatively thinner LMPR compared to human knees [13]. However, the MFL in quadrupeds is analogous to the human posterior MFL [14]. This variability can result in differences in femorotibial contact mechanics and peak pressure values. Nonetheless, previous research on 28 human cadaveric knees investigated the MFL to determine its incidence, structural characteristics, and material properties [14]. This study highlighted the significant anatomical and biomechanical role of the MFL in knee function and concluded that these findings should be considered when evaluating MFL function [14]. It is evident that the MFL plays a critical role in knee stability, regardless of whether the data originate from porcine or human cadaveric models. The next important step is to translate these findings into clinical practice and determine the true relevance of this small but crucial structure in the context of lateral meniscus root surgery.

Additional limitations include the fact that this review focuses on time‐zero cadaveric studies, which inherently do not account for the dynamic stabilisers of the knee, such as muscles and soft tissues, which contribute to joint stability in vivo. Furthermore, the stabilising role of the MFL and the impact of root repair are likely to vary depending on knee flexion angles and tibial movements, factors not fully captured in these studies.

The primary strength of this investigation lies in its clinical relevance. The current systematic review highlights a potential clinical consideration that the MFL should be examined in all cases of LMPR tears, as LMPR tears with intact MFLs may have a less detrimental effect on knee biomechanics than LMPR tears with MFL injuries, especially in cases of concomitant cruciate pathology. Currently, patients are often treated without routine assessment of concomitant MFL injuries, despite their potential clinical significance. The principal contribution of this review lies in its focus on raising awareness of MFL injuries among knee preservation surgeons and researchers. The findings of this study should not provide a definitive rationale for the surgical repair or augmentation of MFL injury in LMPR tears with concomitant ACL injuries. The hypothesis that LMPR repair in ACL‐reconstructed knees may not provide adequate stability is a clinically relevant concern that warrants further investigation using large clinical datasets. While the current meta‐analysis generates hypotheses, definitive conclusions require high‐quality clinical studies. At this stage, the findings are based solely on biomechanical evidence, emphasising the need for further clinical research to validate this hypothesis and facilitate its translation into clinical practice. Through this review, the authors aim to inspire future studies investigating the clinical implications of MFL injuries in the context of LMPR tears, in both MFL‐deficient and intact scenarios. This effort has the potential to open a new chapter in meniscal root preservation surgery by addressing unresolved questions and filling critical gaps in the current literature. By advancing understanding in this area, the authors seek to establish a foundation for better diagnostic and treatment strategies, which represent the primary significance and ultimate goal of this review.

## CONCLUSIONS

A combined injury to the LMPR and MFL significantly increases mean and peak contact pressures in the lateral compartment compared to isolated LMPR tear and intact states, with LMPR repair restoring contact pressure to near‐normal levels. However, in knees with ACL deficiency or reconstruction, LMPR tear with MFL injuries significantly increases IR with pivot shift testing, with LMPR repair unable to restore rotational stability to intact‐state levels.

## AUTHOR CONTRIBUTIONS

**Lika Dzidzishvili**: Substantial conception/design of work, performed data collection, interpretation of data, drafting the work, critically revising the work, manuscript preparation, approving final version for publication, and agreement for accountability of all aspects of work. **Andrew S. Bi**: interpretation of data, revised the manuscript, approving final version for publication, and agreement for accountability of all aspects of work. **Marko Ostojic**: Critically revised the work, approved the final version for publication, and agreement for accountability of all aspects of work. **Jorge Chahla**: Revised the work for important intellectual content, provided final approval of the version for publication, and agreement for accountability of all aspects of work.

## CONFLICT OF INTEREST STATEMENT

Lika Dzidzishvili and Marko Ostojic serve on the Basic Science Committee of the European Society of Sports Traumatology, Knee Surgery, and Arthroscopy (ESSKA). Jorge Chahla reports a relationship with American Orthopaedic Society for Sports Medicine (AOSSM): Arthroscopy Association of North America (AANA): International Society of Arthroscopy, Knee Surgery, and Orthopaedic Sports Medicine (ISAKOS): Board or committee member. Paid consultant, presenter or speaker of Arthrex, CONMED Linvatec, and Smith & Nephew.

## ETHICS STATEMENT

Given the nature of our research, which is a systematic review, ethics approval was not required as it does not involve direct interaction with human subjects or animals. The current study does not involve direct interaction with human subjects, patient consent was not obtained.

## Data Availability

All data supporting the findings of this study are derived from publicly available sources. The datasets analysed during the current study are available in PubMed, Embase, and the Cochrane Library, and were originally published in various medical journals. These sources were accessed to compile and analyse existing research findings relevant to our study.
